# Protein kinase C iota (PKCι) and pVHL are both needed for lysosomal degradation of α5 integrin in renal carcinoma cells

**DOI:** 10.1007/s11033-025-10272-1

**Published:** 2025-01-30

**Authors:** Alissa F. Schurr, Chandni S. Dave, Prachi J. Shah, Jennifer L. Meth, Alexandria S. Jaramillo, Kelly Bartley, Alan R. Schoenfeld

**Affiliations:** Department of Biology, Adelphi University, One South Avenue, P.O. Box 701, Garden City, NY 11530-0701 USA

**Keywords:** Atypical protein kinase C, PKC iota, Von-Hippel Lindau (VHL), α5 integrin, Tight junctions, Lysosomal degradation

## Abstract

**Background:**

von Hippel-Lindau (VHL) hereditary cancer syndrome is caused by mutations in the VHL tumor suppressor gene and is characterized by a predisposition to form various types of tumors, including renal cell carcinomas, hemangioblastomas, and pheochromocytomas. The protein products of the VHL gene, pVHL, are part of an ubiquitin ligase complex that tags hypoxia inducible factor alpha (HIF-α) for proteosomal degradation. pVHL has also been reported to bind to atypical protein kinase C (aPKC).

**Methods and results:**

To better understand the relationship between pVHL and aPKC, the PKC iota (PKCι) isoform of aPKC was knocked out in renal carcinoma cells, both pVHL-negative and those with replaced pVHL. Cellular properties associated with pVHL function were assayed. Knockout of PKCι in pVHL-expressing cells led to greater downregulation of HIF-α than seen with pVHL alone, suggesting that the presence of PKCι opposes complete regulation of HIF-α by pVHL. In contrast, absence of either pVHL or PKCι disrupted tight junction formation and led to upregulated levels of α5 integrin, both of which were phenocopied by lysosomal inhibition. LAMP1 (lysosome associated membrane protein 1), a marker for lysosomes, showed dysregulated localization and altered electrophoretic gel migration in the absence of pVHL. While the upregulated α5 integrin seen in the absence of either pVHL or PKCι loss was associated with increased cell adhesion, loss of pVHL caused increased cell motility whereas loss of PKCι decreased motility.

**Conclusions:**

These data are consistent with a known role of PKCι in endocytosis of α5 integrin and suggest a subsequent novel role of pVHL in targeting a pool of endocytosed α5 integrin for lysosomal degradation.

**Supplementary Information:**

The online version contains supplementary material available at 10.1007/s11033-025-10272-1.

## Introduction

von-Hippel Lindau (VHL) disease, a familial cancer syndrome that predisposes individuals to clear cell renal carcinoma as well as other tumor types, including hemangioblastomas and pheochromocytomas, is caused by inherited mutations in the VHL tumor suppressor gene [1]. The VHL gene produces two proteins that exist in cells and are capable of tumor suppression, VHLp30, which is translated from the first start codon, and VHLp19, which is translated internally from the second start codon [2–4]. Either of these VHL proteins (collectively called pVHL) can be a component of an E3 ubiquitin ligase complex that binds substrates, which are then ubiquitinated and subsequently targeted for degradation [5, 6]. The most commonly known target of pVHL is hypoxia-inducible factor alpha (HIF-α, the collective name of the gene family including HIF-1α and HIF-2α), a subunit of the heterodimeric HIF transcription factor [7–9]. HIF upregulates hypoxia-inducible genes in conditions of low oxygen, when pVHL is unable to bind to HIF-α, and in normoxic conditions when VHL is mutationally or epigenetically inactivated [7]. Ubiquitination of HIF-α is considered to be the most important tumor suppressing activity of pVHL, especially since removal of HIF-α is sufficient to inhibit the tumorigenicity of pVHL-deficient renal cell carcinoma cells [10, 11].

In addition to binding to HIF-α, pVHL has been reported to associate with atypical protein kinase C (aPKC) [12]. aPKC is a subgroup of the protein kinase C (PKC) family of serine-threonine kinases that are involved in signal transduction (reviewed in [13]). aPKC differs from the other PKC subgroups in that it is not activated by diacylglycerol and/or Ca^+2^ binding, but instead requires PAR6 and CDC42 to interact with its PB1 (Phox and Bem 1) domain (reviewed in [13]). There exists two isoforms of aPKC, PKC iota (PKCι, known as PKCλ in mice) and PKC zeta (PKCζ). Both of these isoforms were shown to bind to the β-domain of pVHL, which is the same region that HIF-α binds to [12]. PKCλ was subsequently shown to be ubiquitinated by the pVHL complex in vitro and in vivo in HEK293T cells that were transiently transfected with PKCλ [14]. In these studies, a PKCλ mutant with four arginines substituted with alanines in the pseudosubstrate domain [15], indicated as the active form of PKCλ, was preferentially ubiquitinated by pVHL, leading to the conclusion that activated aPKC is the substrate of pVHL-mediated ubiquitination [14]. Unlike with HIF-α, overall aPKC levels were not affected by pVHL [14]. In agreement with pVHL-mediated degradation of active aPKC, levels of a slower migrating endogenous aPKC band (consistent with phosphorylated, active aPKC) decreased upon wild-type pVHL expression in pVHL-null 786-O cells [16].

The cellular functions of aPKC include regulation of tight junction formation, cell polarity, integrin expression and adhesion, directional migration, and cell morphology ( [17–19], also reviewed in [13, 20]). Interestingly, most of these activities have also been associated with pVHL function [21–25]. Moreover, loss of pVHL expression in *Drosophila* follicular epithelium has previously been shown to cause mislocalization and decreased stability of aPKC, along with loss of epithelial polarity, a key role of aPKC, although likely occurring indirectly through defects in pVHL-mediated microtubule formation [26]. The necessity of pVHL for proper aPKC activity could suggest that they function cooperatively with each other such that the absence of one of these proteins may result in a hindrance of their related functions. To investigate this possibility and further elucidate the functional relationship between pVHL and aPKC, a CRISPR-Cas9 genome editing approach was implemented to produce a knockout of PKCι in renal cell carcinoma cells lacking pVHL and with pVHL reintroduced. Functional assays were conducted to investigate the effects of the PKCι knockout on these cells. Herein, we describe distinct roles of PKCι that interact with pVHL cellular functions.

## Methods

### Cell culture

786-O renal carcinoma cells were obtained from the American Type Culture Collection. RCC10 renal carcinoma cells were generously provided by Miguel Esteban (Imperial College, London). All cells were grown in Dulbecco’s modified Eagle’s medium (DMEM), supplemented with 10% fetal bovine serum (Atlas Biologicals, Fort Collins, CO) and 1% penicillin-streptomycin (100 U/ml and 10 µg/ml, respectively). 786-O and RCC10 cell lines containing stably reintroduced VHLp19 had been created previously [2, 25].

### CRISPR plasmid construction

The lentiCRISPR v2 plasmid [27] was a gift from Feng Zhang (Addgene plasmid # 52961; http://n2t.net/addgene:52961; RRID: Addgene_52961). LentiCRISPRv2 plasmid was digested with *BsmBI* restriction enzyme and ligated with annealed sgRNA oligonucleotides as described in the Target Guide Sequence Cloning Protocol found at the Addgene website provided. sgRNA sequences targeting PKCι were TGTCTCGAACCTCATTGCAA (exon 2, antisense strand) and TCCAAGCCAAGCGTTTCAAC (exon 5, sense strand), hereafter named PKCι sgRNA 1 and 2, respectively. sgRNA sequence targeting PKCζ was CCATCCATCCCATCGATAAC (exon 9, antisense strand).

### Transfection and selection of CRISPR 786-O clones

Lipofectamine was used to transfect 786-O cells (both parental and VHLp19-expressing) with LentiCRISPRv2 plasmids containing either PKCι sgRNA 1 or 2 or containing an sgRNA targeting the *E. Coli* β-galactosidase (LacZ) gene (TGCGAATACGCCCACGCGAT), as a control. VHLp19-expressing RCC10 cells were similarly transfected with PKCι or PKCζ (or LacZ) sgRNA plasmids. Following the transfections, cells were re-plated at a lower confluency in 100 mm culture dishes and selected with puromycin (1 µg/ml) at 3 days post-transfection. Approximately 2–3 weeks after selection, colonies were isolated with cloning cylinders and expanded. Knockouts (or knockdowns) were identified by western blotting, with PKCι knockouts further verified by DNA sequencing (GenBank accession numbers MZ367579, MZ367580, MZ367581, MZ367582, MZ367583, MZ367584, MZ367585, and MZ367586).

### Western blotting

Cells in 60 mm culture dishes were rinsed with PBS and then lysed by incubating with 150 µl of lysis buffer (50 mM HEPES (pH 7.6), 250 mM NaCl, 0.5% Nonidet P-40, 0.5% Triton X-100, 5 mM EDTA, 1mM phenylmethylsulfonyl fluoride (PMSF), 1 mM Na_2_VO_3_ and 2 µg/ml each of aprotinin, bestatin, and leupeptin) at 4 °C for 30 min. Lysates were collected and spun down in a refrigerated microcentrifuge for 15 min to remove all insoluble material. The supernatant was collected and a Bradford protein assay (Bio-Rad, Hercules, CA) was performed to determine protein concentrations. Equal amounts of protein for each well of a gel, ranging from 25 to 50 µg among the different blots performed, were mixed with an equivalent volume of 2× SDS buffer and were separated by SDS-PAGE. The separated proteins were then transferred to a polyvinylidene difluoride (PVDF) membrane overnight at 30 volts for 16 h. Western blotting was performed using 5% bovine serum albumin (BSA) in TBS-Tween (20 mM Tris, pH 7.8; 150 mM NaCl; 0.5% Tween-20) to both block the membrane and dilute primary antibodies. Secondary antibodies were diluted in 1% nonfat dry milk (Bio-Rad, catalog (cat.) #1706404) in TBS-Tween. Bands were visualized via chemiluminescence using ECL Western Blotting Substrate (Pierce, cat. # 32106) and standard x-ray film and developer. Band intensities were quantified using Gel Analyzer in ImageJ (version 1.54) and the intensities were divided by their corresponding α-tubulin control intensities (from the same lane and from the same gel), and then normalized with the first (control) lane of each blot set to 100%. When replicates were presented, Student’s t-tests (two sample unequal variance) were used for pairwise statistical comparisons.

### Antibodies used for western blotting

PKCι and PKCζ rabbit antibodies (cat. #s sc-17837 and sc-216, Santa Cruz Biotechnology, Santa Cruz, CA) were used at a 1:200 dilution in western blots. pVHL rabbit antibody (cat. # 68547, Cell Signaling Technology, Danvers, MA) was used at a 1:1000 dilution. HIF-2α rabbit antibody (cat. # NB100-122, Novus Biological, Littleton, CO) was used at a 1:500 dilution. α5 integrin and β1 integrin mouse monoclonal antibodies (cat. #s 610633 and 610467, BD Biosciences, Franklin Lakes, NJ) and LAMP1 rabbit monoclonal antibody (cat. # 9091, Cell Signaling Technology) were used at a 1:1000 dilution. α-tubulin mouse monoclonal antibody (cat. # T6074, Sigma, St. Louis, MO) was used at a 1:2000 dilution. Anti-mouse IgG-HRP and anti-rabbit IgG-HRP secondary antibodies (cat. #s 1031-05 and 4050-05, Southern Biotech, Birmingham AL) were used at a 1:2286 dilution.

### Immunofluorescence microscopy

Confluent cells grown on coverslips in a 6-well plate were washed twice with PBS and were then fixed and permeabilized by a 1 min incubation in a 1:1 mixture of methanol/acetone (−20 °C). Cells were then rinsed three times with PBS (for 3 min each) and incubated at room temperature in 1 ml of blocking solution (1% goat serum (cat. # 0061−01, Southern Biotech, Birmingham, AL), 0.1% Tween-20 in PBS) for 50 min. For zona occludens-1 (ZO-1) immunostaining, cells were then incubated in ZO-1 rabbit antibody (cat. # 40–2200, Zymed/Invitrogen), diluted 1:90 in blocking solution, for 50 min. The cells were washed three times for 5 min each with PBS-Tween-20 on a rocking platform. 75 µl of anti-rabbit-TRITC secondary antibody (cat. # 4050-03, Southern Biotech) at a 1:100 dilution in blocking solution was added to each coverslip, which were incubated at room temperature for 1 h while covered by aluminum foil. The cells were washed 3 times as previously except that during the second wash, 4’,6-diamidino-2-phenylindole (DAPI, 0.25 µg/ml) was added. The coverslips were mounted on slides with GelMount and viewed at a magnification of 1000× on a Zeiss Axioskop microscope, with images collected by a Spot Insight QE digital camera. Random fields containing approximately equal numbers of nuclei (to eliminate any possible effects of confluency) were selected for viewing and exposures during imaging were kept constant for all samples (10 s and 0.2 s for ZO-1 and DAPI, respectively) to allow for more direct comparisons. For LAMP1 immunostaining, cells were handled as previously described for ZO-1 staining, except Alexa Fluor 488-conjugated LAMP-1 rabbit monoclonal antibody (cat. # 58996, Cell Signaling Technology) was used at a 1:100 dilution (with no secondary antibody steps needed).

### Cycloheximide and bafilomycin assays

For both assays, cell lines were grown to confluence in 60 mm culture dishes. For cycloheximide assays, cells were then either left untreated or treated with 100 µg/ml cycloheximide (cat. # C7698, Sigma, St. Louis, MO) for various time points and then lysed and assayed by western blotting. For bafilomycin assays, cells were treated overnight either with vehicle (DMSO) or with 10 nM Bafilomycin A1 (cat. # B1793, Sigma, St. Louis, MO).

### Adhesion assay

Cells were plated in 96 well plates at a density of 5 × 10^4^ cells per well, with each cell line plated in sextuplicate (2 sets of triplicates), and allowed to grow overnight. Wells for one set of triplicates of each cell line were washed with PBS and incubated in 10 mM EDTA in PBS with no Ca^+2^ and Mg^+2^ for 1 h at room temperature. Wells were then agitated to release suspended cells by pipetting up and down 3 times with a multichannel pipette and then aspirated. The other set of triplicates remained untreated up to this point. Wells from both sets of triplicates were rinsed with 100 µl of PBS, fixed with 50 µl of fixing solution (10% acetic acid/10% methanol) for 15 min, and then stained with 30 µl of 1% crystal violet in methanol for 10 min. Crystal violet stain was aspirated, and wells were washed three times with 200 µl of water. Crystal violet stained cells were solubilized by incubating for 10 min in 10% acetic acid and absorbances of these samples were read by microplate reader (Bio-Rad) at a wavelength of 590 nm. Readings from untreated cells of each colony were used to normalize the EDTA-treated wells. For cells with LacZ and PKCι sgRNA, two independently-isolated clonal lines were assayed and their results were averaged. ANOVA and Tukey’s post-hoc tests were performed using SPSS statistical software.

### Scratch-wound assay

Wounds were created by dragging the tip of a cell scraper through 60 mm plates of confluent cells to clear lines of cells through the monolayer. Three wounds were created for each plate, with lines previously drawn on the plate perpendicular to the wound to allow imaging of the exact same location of each wound. Digital images of wounds were taken immediately after wounding and 7 h later. The area of each wound was determined from the images using ImageJ (National Institutes of Health) and the area filled in for each wound was calculated. 9 wounds for each cell line were averaged. ANOVA and Tukey’s post-hoc tests were performed using SPSS statistical software.

## Results

### Knockout of PKCι leads to VHL-independent downregulation of HIF-2α levels

**Fig. 1:**
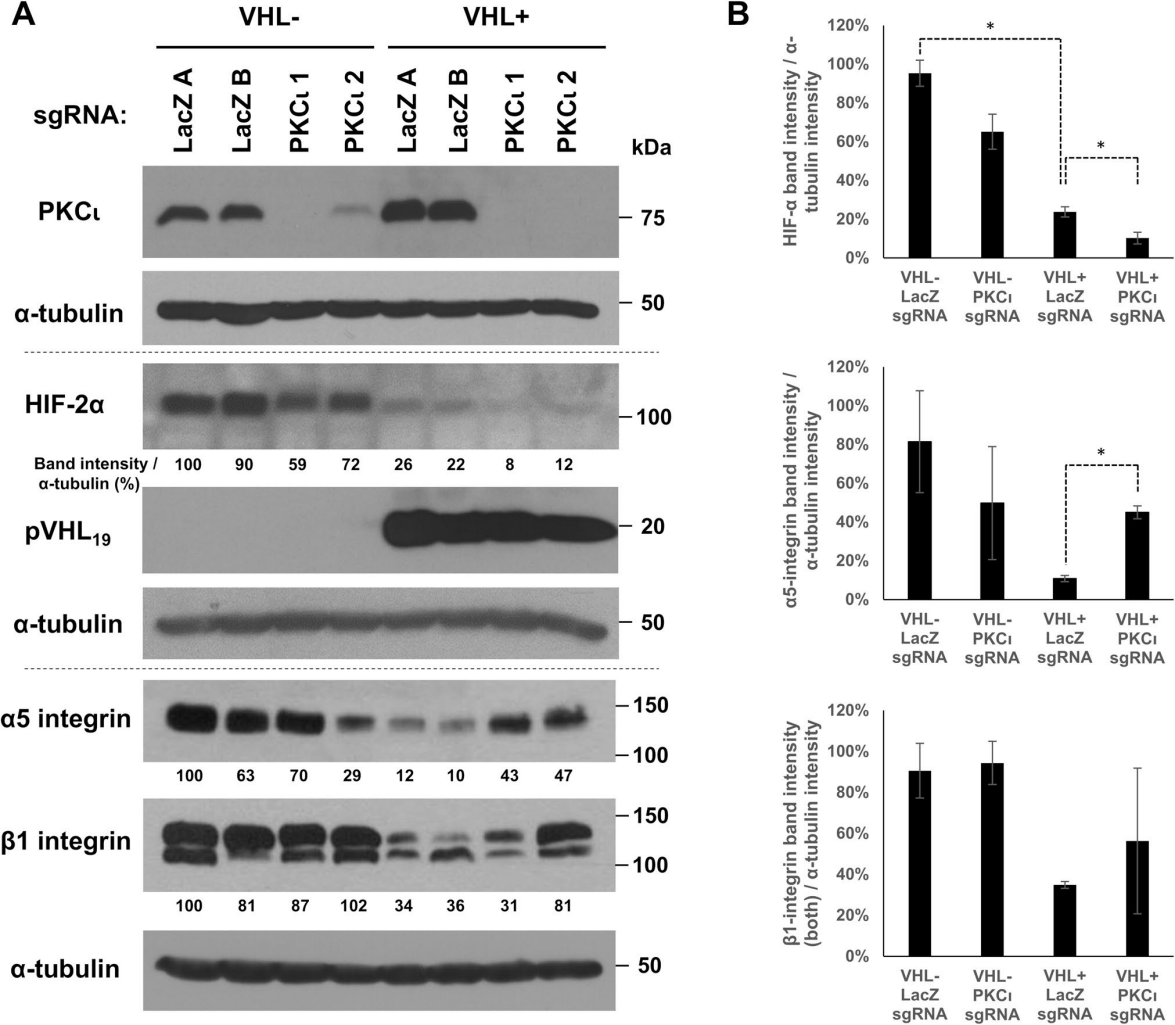
Knockout of PKCι in 786-O cells potentiates pVHL’s downregulation of HIF-2α but abrogates pVHL-mediated downregulation of α5 integrin levels. CRISPR-Cas9 targeting of parental 786-O cells (VHL-) and 786-O cells with reintroduced VHLp19 (VHL+) was performed. Two colonies targeting LacZ as a control (each with the same LacZ sgRNA, designated A and B), and two colonies targeting PKCι (each with different PKCι sgRNAs, PKCι 1 and PKCι 2) were isolated for each cell type (VHL- or VHL+) and assayed by western blot, as indicated. (**A**) Western blots from separate experiments are shown together, with horizontal dotted lines separating them. Western blot was performed using an antibody specific to the PKCι isoform (top panel). The membrane was then reblotted with an α-tubulin antibody as a loading control (2nd panel). Lysates derived from the same cells as in the top two panels were used to perform western blots using antibodies specific to HIF-2α (3rd panel) and pVHL (4th panel), and then reblotted for α-tubulin (5th panel), as indicated. Lysates derived from those same cells were used to perform western blots using antibodies specific to α5 integrin (6th panel) and β1 integrin (7th panel), and then reblotted for α-tubulin (8th panel), as indicated. Band intensities were divided by their corresponding α-tubulin band intensities (from the same lane) and presented as a percent, with the first lane set to 100%. (**B**) Graphical representation of band intensities for HIF-2α, α5 integrin, and β1 integrin, with averages of cell lines with the same sgRNA targeting/VHL status shown, with standard deviations as the error bars. Student’s t-tests were used for pairwise statistical comparisons. * indicates statistical significance (*P* < 0.05)

To better elucidate the functional relationship between pVHL and aPKC, CRISPR-Cas9 expression plasmids were used to produce knockouts of PKCι in both parental 786-O cells and those with reintroduced pVHL. Two different single guide RNAs (sgRNAs) targeting PKCι were used (PKCι 1 and PKCι 2) targeting exons 2 and 5, respectively, to reduce off-target effects, and colonies derived from single-cells were isolated. Colonies with CRISPR-Cas9 constructs targeting *E. coli* β-galactosidase (LacZ) were also generated and used as negative controls. Despite a large number of colonies assayed, only a few colonies demonstrated PKCι knockout or in the case of one clone in pVHL-null 786-O cells, knockdown (most likely as a result of a CRISPR-mediated event occurring in a single allele of PKCι). The colonies shown in Fig. 1A (top panel) were chosen for further functional analysis.

Western blots for pVHL and HIF-2α were performed (Fig. 1A, 3rd and 4th panels). HIF-1α was not assayed as it is not measurably expressed in 786-O cells [7]. Loss of PKCι had no noticeable effect on pVHL levels (in the pVHL-expressing cells). Interestingly, HIF-2α levels were slightly but noticeably decreased in the pVHL-null cell line with PKCι knockout (Fig. 1A, HIF-2α panel, compare lane 3 to lanes 1–2). As expected, pVHL expression led to robust downregulation of HIF-2α (Fig. 1A, HIF-2α panel, compare lanes 5–6 to 1–2) that was statistically significant (Fig. 1B, top graph). In the pVHL-expressing cells, knockout of PKCι led to even lower expression of HIF-2α as compared to the LacZ sgRNA controls (Fig. 1, HIF-2α panel, compare lanes 7–8 to 5–6) that was also statistically significant (Fig. 1B, top graph). Thus, the presence of PKCι in 786-O cells seemed to hinder a more complete removal of HIF-2α by pVHL.

### Knockout of PKCι abrogates pVHL-mediated tight junction formation and downregulation of α5 integrin levels

**Fig. 2:**
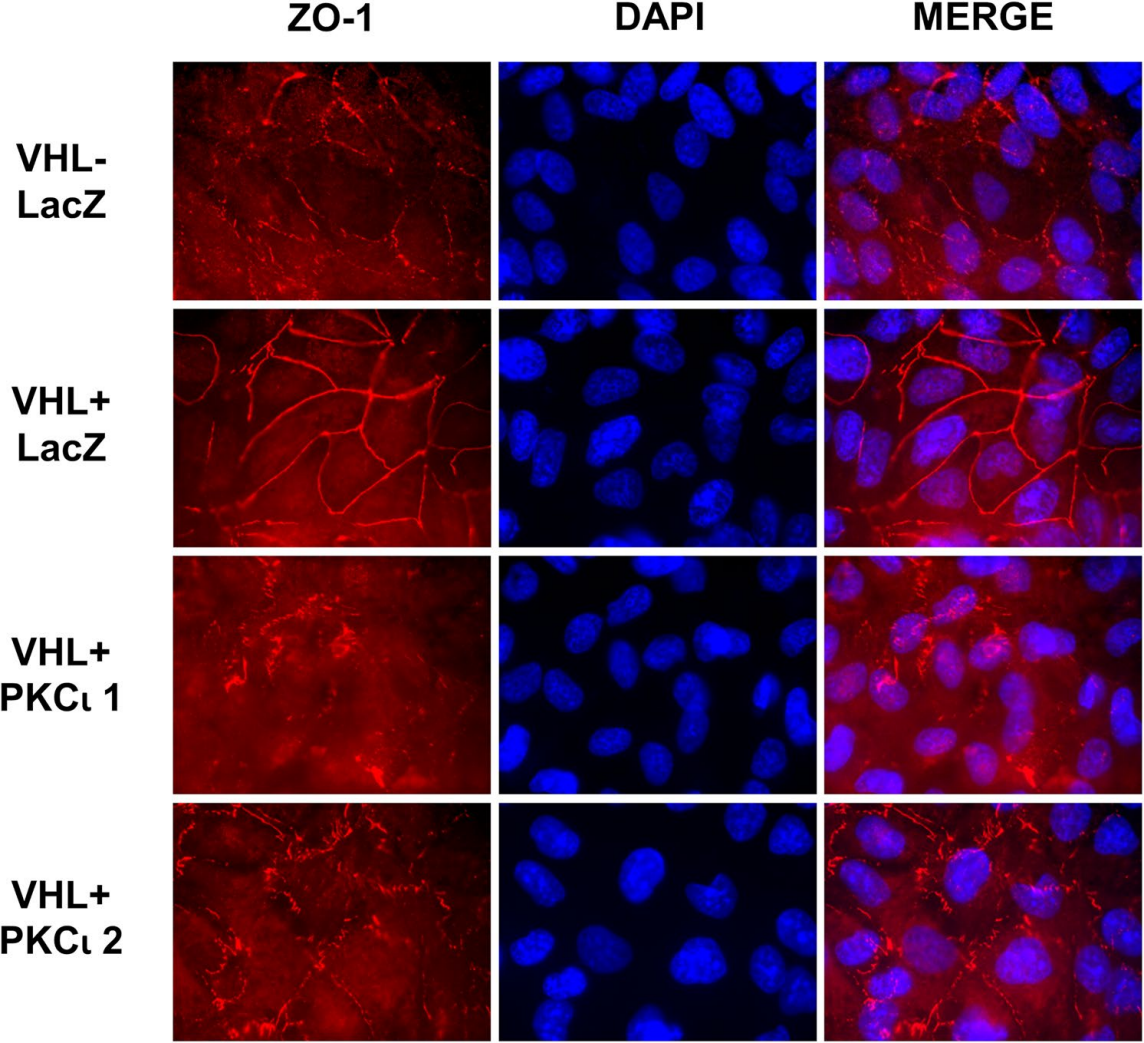
Knockout of PKCι in pVHL-expressing 786-O cells disrupts tight junction formation. 786-O cells without pVHL (1st row) and with pVHL reintroduced (rows 2–4) were grown on coverslips to confluence and assayed by indirect immunofluorescence staining for ZO-1 as a tight junction marker. Left panels show ZO-1 localization, center panels show stain for DAPI (nuclei), and the right panel depicts a merged image of the ZO-1 and DAPI stains. Cell lines used and their sgRNA targets are indicated on the left of each row. The images were taken at 1000× magnification

pVHL expression has been shown to be important for the formation of tight junctions in renal cells [22, 23, 25]. To examine the necessity for PKCι in the pVHL-associated regulation of tight junctions, immunostaining was performed, looking at ZO-1 as a marker of tight junctions. As previously, pVHL-null cells (with LacZ control sgRNA) revealed a disorganized pattern of ZO-1 localization (Fig. 2, top row), whereas pVHL-expressing 786-O cells (with LacZ control sgRNA) showed crisp lines of ZO-1 localized to the cell borders (Fig. 2, 2nd row). However, ZO-1 localization and presumably tight junction formation was noticeably disrupted in cells in both PKCι knockout cell lines (Fig. 2, bottom two rows). These results imply that the pVHL influence on tight junction formation cannot occur in the absence of PKCι.

Since both aPKC and pVHL have an effect on integrins, western blots for α5 and β1 integrin were performed (Fig. 1A, 6th and 7th panels). As has been seen previously [21, 25], pVHL expression caused reduced levels of these integrins (in the VHL + cells with LacZ sgRNA) (Fig. 1A, 6th and 7th panels, compare lanes 1–2 to 5–6). Of note, PKCι knockout in the pVHL-expressing cells led to increased levels of α5 integrin, approaching levels seen in the pVHL-null cells (Fig. 1A, α5 integrin panel, compare lanes 7–8 to 5–6). This increase was statistically significant (Fig. 1B, middle graph). Unlike the VHL-expressing cells, PKCι knockout in the pVHL-null cells did not further increase levels of α5 integrin (Fig. 1A, α5 integrin panel, compare lane 3 to lanes 1–2), although the PKCι knockdown showed decreased levels in one of the two lines (PKCι 2, Fig. 1A α5 integrin panel, lane 4) for reasons that are not immediately evident. Overall, the increased α5 integrin in pVHL-expressing PKCι knockout lines may suggest that PKCι is needed for pVHL-mediated downregulation of α5 integrin.

Interestingly, while α5 integrin showed a clear upregulation in VHL-expressing cells with PKCι removed, the pattern for β1 integrin was similar but not as uniform (Fig. 1A, 7th panel, compare lanes 7–8 to 5–6). There was a more noticeable upregulation of β1 integrin in the PKCι 2 knockout line (Fig. 1A, β1 integrin panel, compare lane 8 to lanes 5–6) that was not observed to the same degree in the PKCι 1 line (Fig. 1A, β1 integrin panel, lane 7). There also was an apparent shift to a higher proportion of the slower migrating form of β1 integrin in the VHL-expressing PKCι knockouts (Fig. 1A, β1 integrin panel, compare lanes 7–8 to 5–6), which likely represents a shift from immature to mature forms of β1 integrin [28, 29]. Because the upregulation of β1 integrin did not occur to the same magnitude in both VHL-expressing PKCι knockout cell lines and was not statistically significant (Fig. 1B, bottom graph), the focus herein will be on the changes to α5 integrin, which did.

### Knockout of PKCι in pVHL-expressing RCC10 cells corroborates downregulation of HIF-2α and abrogation of pVHL-mediated downregulation of α5 integrin

**Fig. 3:**
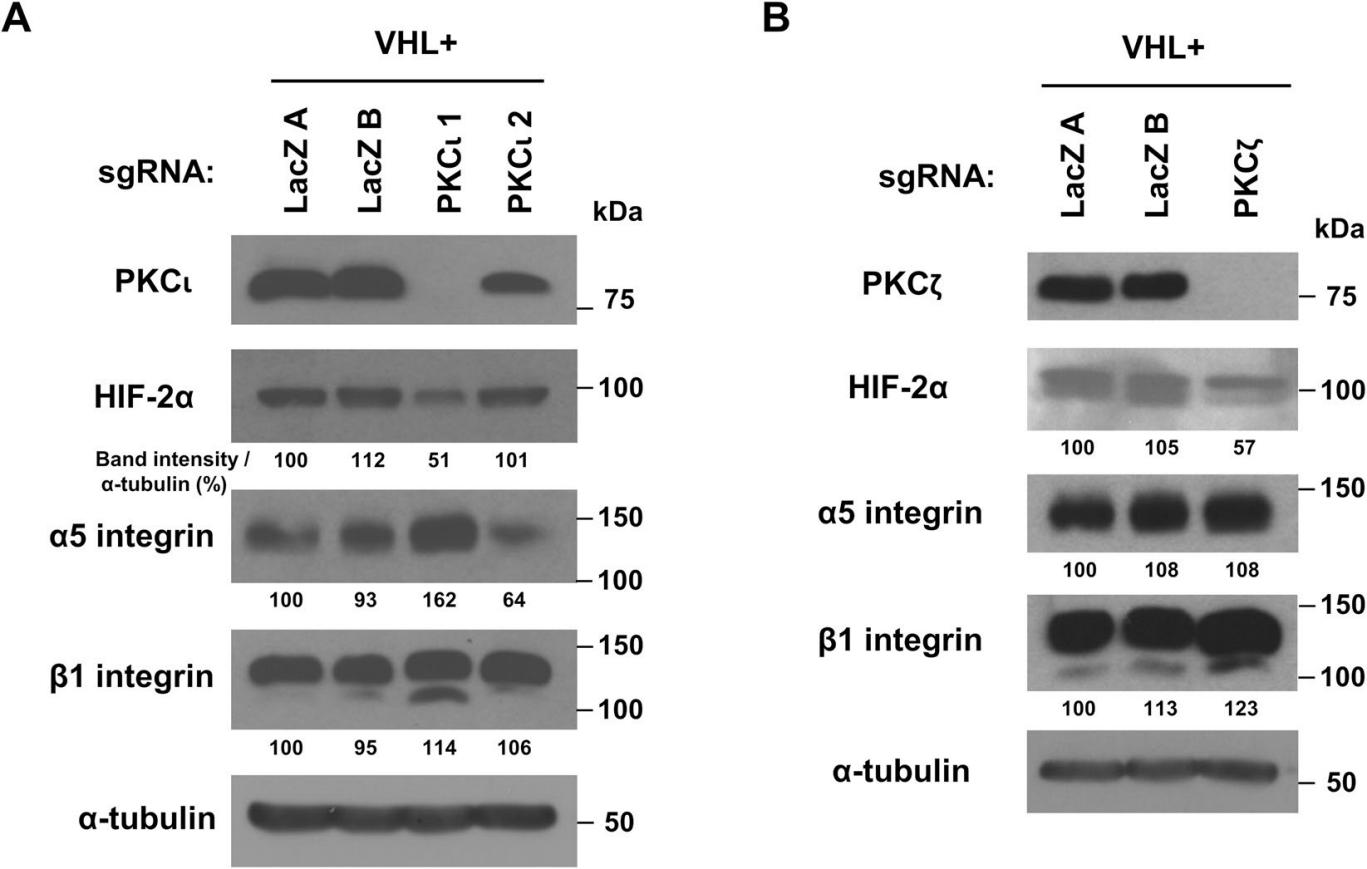
Knockout of PKCι in RCC10 cells decreases levels of HIF-2α and abrogates pVHL-mediated downregulation of α5 integrin levels. (**A**) A western blot of lysates from colonies of RCC10 cells with reintroduced VHLp19 (VHL+) was performed. Two colonies targeting Lac Z (each with the same LacZ sgRNA, designated A and B) and two colonies targeting PKCι (each with different PKCι sgRNAs, PKCι 1 and PKCι 2) were assayed. Western blot was performed using an antibody specific to the PKCι isoform (top panel). Western blots were performed on gels/blots processed in parallel using the same samples using antibodies specific to HIF-2α (2nd panel), α5 integrin (3rd panel), β1 integrin (4th panel), and α-tubulin (as a loading control, on a reblotted membrane, 5th panel), as indicated. Band intensities were divided by their corresponding α-tubulin band intensities (from the same lane) and presented as a percent, with the first lane set to 100%. (**B**) Similar analyses as in (A), but with RCC10 cells in which PKCζ was targeted by a sgRNA, as well as 2 control cell lines in which LacZ was targeted. All samples derive from the same experiment using gels/blots that were processed in parallel

To ensure that the downregulation of HIF-2α and upregulation of α5 integrin in pVHL-expressing cells seen upon PKCι knockout were not particular to the 786-O cell line, CRISPR-Cas9-mediated knockout of PKCι in RCC10 renal carcinoma cells was performed on pVHL-expressing cells. One knockout was obtained using PKCι sgRNA 1 and a possible knockdown was seen with PKCι sgRNA 2 (Fig. 3A, top panel), although the focus here will be on the complete knockout (lane 3 in all parts of Fig. 3A; compare to controls in lanes 1 and 2). Consistent with 786-O, lower levels of HIF-2α were observed in the PKCι knockout (Fig. 3A, 2nd panel) and increased α5 integrin expression was also seen with this RCC10 clone (Fig. 3A, 3rd panel). To see if loss of PKCζ causes similar effects, PKCζ was knocked out in RCC10 cells (Fig. 3B). While slightly lower levels of HIF-2α were observed in the PKCζ knockout (Fig. 3B, 2nd panel), levels of α5 integrin were not increased in this PKCζ knockout line (Fig. 3B, 3rd panel), indicating that regulation of α5 integrin is a property more associated with the PKCι isoform.

### pVHL-mediated downregulation of α5 integrin requires lysosomal function

**Fig. 4:**
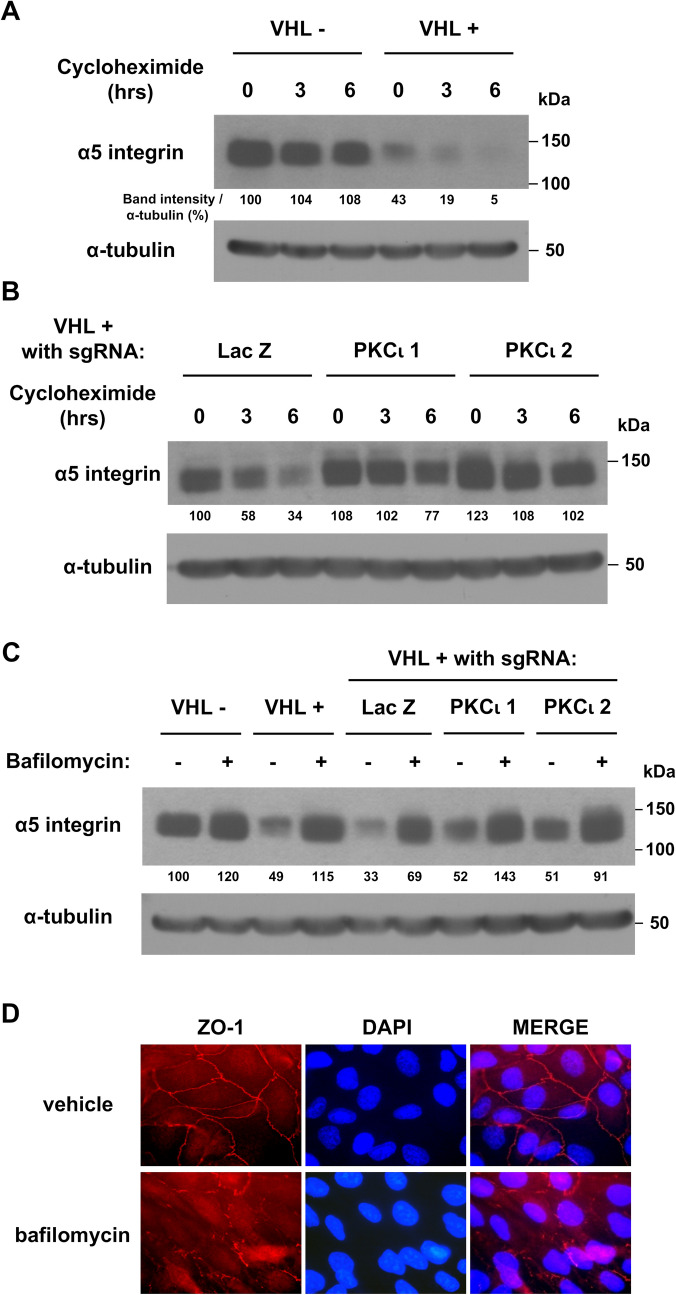
Cycloheximide and bafilomycin assays reveal that pVHL-mediated downregulation of α5 integrin levels requires lysosomal degradation. (**A**) Parental 786-O cells (VHL-) and those with reintroduced VHLp19 (VHL+) were incubated with cycloheximide (100 µg/ml for various time points and then a western blot for α5 integrin was performed, with an α-tubulin western blot as a loading control. Band intensities were divided by their corresponding α-tubulin band intensities (from the same lane) and presented as a percent, with the first lane set to 100%. (**B**) Similar analysis was performed as in (A) with 786-O cell lines with pVHL reintroduced (VHL+) containing sgRNA targeting LacZ (as a control) or PKCι (PKCι 1 and PKCι 2). (**C**) 786-O cell lines used in (A) and (B) were incubated with vehicle or bafilomycin (10 nM) overnight and a western blot for α5 integrin was performed, with an α-tubulin western blot performed as a loading control. Note that in (A), (B), and (C), the α-tubulin western blots were performed by reblotting the membranes in the top panel. (**D**) Cells with reintroduced VHLp19 (VHL+) were incubated with DMSO (vehicle) or bafilomycin (10 nM) overnight before ZO-1 immunostaining, as previously described. Images were taken at 1000× magnification

Cycloheximide assays were performed to look at the stability of α5 integrin. Since this assay had not been performed previously on parental 786-O cells and the pVHL-expressing cells on which the sgRNA clones were based, these were included in the assay (VHL- and VHL+, respectively, in Fig. 4A). α5 integrin was much more stable in cells without pVHL than when pVHL was present (Fig. 4A). The colonies of the sgRNA-targeted cells were then analyzed, focusing on pVHL-expressing cells (Fig. 4B), since these showed decreased α5 integrin stability. Lack of PKCι in pVHL-expressing cells led to a rebound in α5 integrin stability as compared to cells expressing PKCι (Fig. 4B, compare PKCι 1 and 2 knockouts to control with LacZ sgRNA). Thus, the presence of both pVHL and PKCι appears to be needed for decreased stability of α5 integrin. Because α5 integrin is regulated by internalization and lysosomal degradation [30], the effects of balifomycin, an inhibitor to the proton pump responsible for lysosomal acidification needed for protein degradation, were tested on the set of 786-O cell lines. Bafilomycin treatment abrogated the downregulation of α5 integrin seen in cells containing pVHL (Fig. 4C, compare lanes 3 to 4 and 5 to 6). Interestingly, cells with a PKCι knockout, which display a level of α5 integrin that is slightly upregulated as compared to LacZ-targeted cells containing both pVHL and PKCι, show a further upregulation of α5 integrin upon bafilomycin treatment (Fig. 4C, lanes 7–8 and 9–10; compare to 5–6). These data suggest that loss of pVHL or, to a somewhat lesser extent, absence of PKCι, hinders lysosomal degradation of α5 integrin. Interestingly, tight junction formation in pVHL-expressing cells was disrupted upon inhibition of lysosomal function by bafilomycin (Fig. 4D), which could be related (at least in part) to the upregulation of α5 integrin caused by this treatment.

### Lack of pVHL increases cell motility, whereas lack of PKCι decreases it

**Fig. 5:**
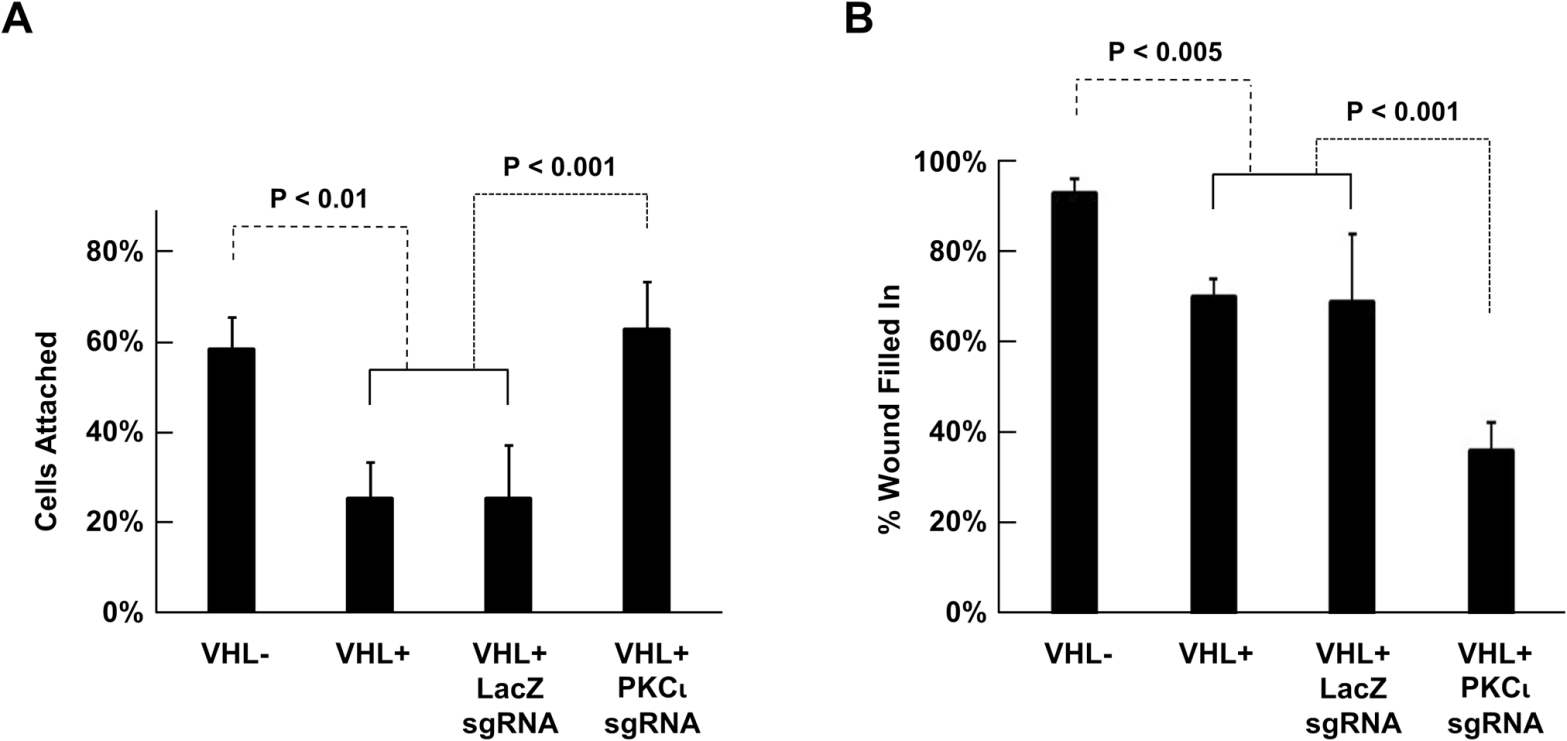
Lack of pVHL and PKCι both increase cell adhesion, but differentially affect cell migration. (**A**) Cell adhesion assay was performed on parental 786-O cells (VHL-) and those with reintroduced VHLp19 (VHL+), as well as colonies of cells with reintroduced VHLp19 containing sgRNAs targeting LacZ and PKCι (PKCι knockout cells), as indicated. For both cells with LacZ and PKCι sgRNA, results from the two clonal lines of each were combined in the graph. Percent of cells attached after EDTA treatment (as described in Materials and methods) was normalized to untreated cells of the same cell type. Cells with VHLp19 reintroduced (VHL+) and those with sgRNA targeting LacZ showed no statistical difference (*P* > 0.99). Parental 786-O (VHL-) showed statistically significant greater attachment than cells with VHLp19 reintroduced and those with sgRNA targeting LacZ (*P* < 0.01 for both). Cells with VHLp19 introduced containing sgRNA targeting PKCι (PKCι knockout cells) showed statistically significant greater attachment than cells with VHLp19 reintroduced and those with sgRNA targeting LacZ (*P* < 0.001 for both). (**B**) Scratch-wound assay performed with the same cells as in (**A**). Cells with VHLp19 reintroduced (VHL+) and those with sgRNA targeting LacZ showed no statistical difference (*P* > 0.99). Parental 786-O (VHL-) showed statistically significant greater mobility than cells with VHLp19 reintroduced and those with sgRNA targeting LacZ (*P* < 0.005 for both). Cells with VHLp19 introduced containing sgRNA targeting PKCι (PKCι knockout cells) showed statistically significant less mobility than cells with VHLp19 reintroduced and those with sgRNA targeting LacZ (*P* < 0.001 for both)

Given the role of integrins in adhesion of cells to substrate, this property was analyzed further. Cell adhesion assays were carried out, looking at the percentage of cells that remained attached after treatment with EDTA to remove divalent cations that are needed for integrin function (Fig. 5A). Again, since this assay was novel for parental 786-O cells and the VHLp19-expressing cells on which the sgRNA clones were based, these were included in the assay (VHL- and VHL+, respectively, in Fig. 5A). After a 1-hour treatment with 10 mM EDTA, the majority of pVHL-null 786-O cells remained attached. Cells with reintroduced pVHL were significantly less attached, as were pVHL-expressing cells with control LacZ sgRNA. However, the majority of the pVHL-expressing cells with PKCι knockout remained attached, to the same extent as parental pVHL-null cells. These results parallel the α5 integrin levels in these cells (with higher α5 integrin associated with stronger adhesion) and suggest that PKCι is needed for the pVHL-mediated decrease in cell adhesion. Since integrins also play an important role in cell migration, a scratch-wound assay was performed on these same cells (Fig. 5B). While pVHL-negative 786-O cells, with higher levels of α5 integrin, were observed to have increased mobility as compared to pVHL-expressing cells (both original and LacZ sgRNA-containing), pVHL-expressing cells with PKCι knocked out, which also have higher levels of α5 integrin, were seen to have decreased mobility. These data suggest that both pVHL and PKCι play a role in lysosomal regulation of α5 integrin such that their loss results in increased levels, but likely through different but perhaps related mechanisms.

### pVHL expression alters LAMP1 localization and glycosylation

**Fig. 6:**
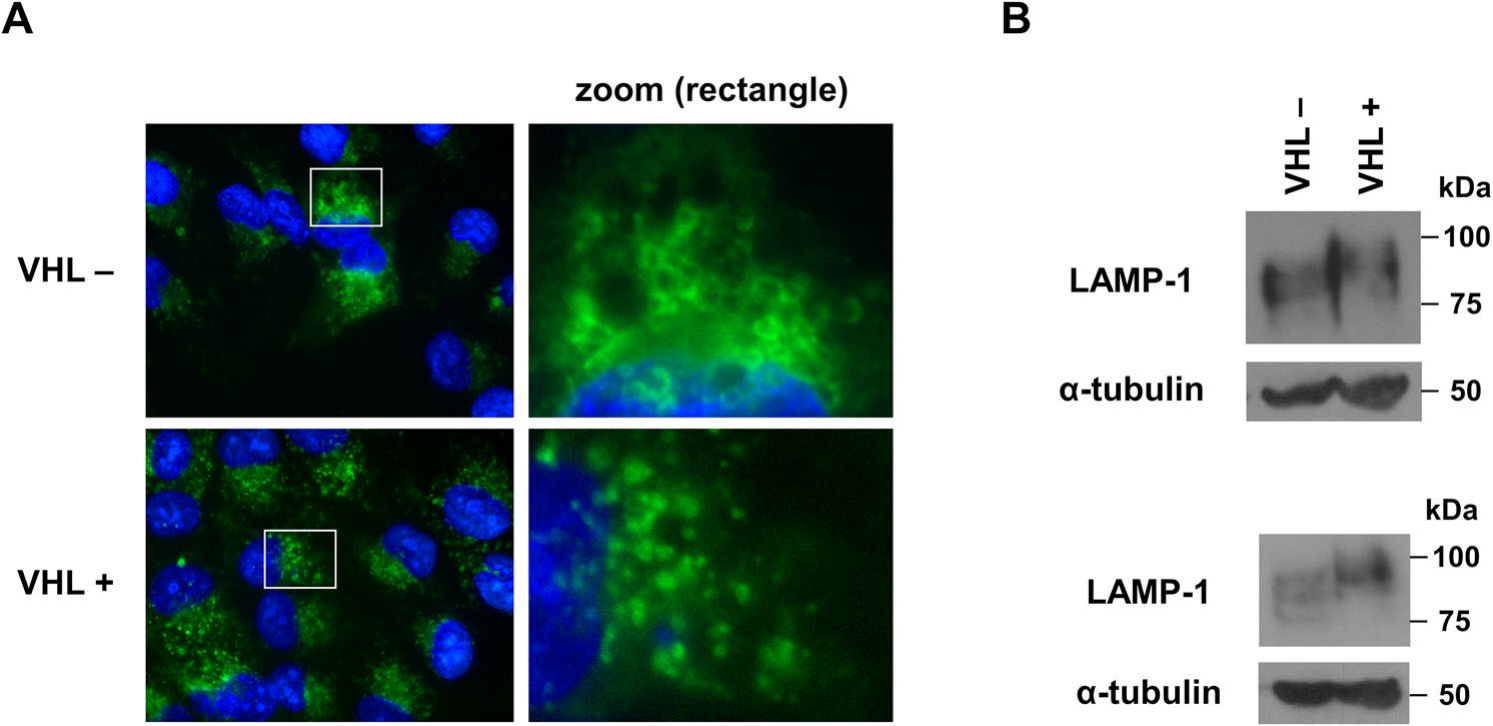
Presence of pVHL leads to altered localization and glycosylation of LAMP1. (**A**) Immunofluorescence for endogenous LAMP1 was performed on parental 786-O cells (VHL-, top row) and those with reintroduced VHLp19 (VHL+, bottom row). Merged images with LAMP1 staining (green) and DAPI co-staining of nuclei (blue) are shown. The images on the left were taken at 1000× magnification. The insets with the white rectangles in the left panels were enlarged by a factor of 5 in the images on the right (zoom). (**B**) A western blot of lysates from colonies of parental 786-O cells (VHL-) and those with reintroduced VHLp19 (VHL+) was performed with LAMP1 antibody. The experiment was repeated with different lysates and both results are presented

It is possible that perturbation of lysosomal regulation of α5 integrin in pVHL-negative cells occurs due to a change in lysosomal function. To investigate any apparent differences in lysosomes between pVHL-negative and pVHL-expressing 786-O cells, immunostaining for endogenous LAMP1, a lysosomal membrane glycoprotein (reviewed in [31]), was performed (Fig. 6A). LAMP1 localized to vesicles that were adjacent to the nucleus, in agreement with what has been described for LAMP1 localization [32]. Interestingly, these vesicles were considerably larger in size in pVHL-negative cells than in pVHL-expressing cells (Fig. 6A, compare top and bottom panels). A western blot for LAMP1 was also performed (Fig. 6B). In pVHL-null cells, LAMP1 was seen as a set of bands from approximately 75 to 90 kDa (Fig. 6B, top). However, in pVHL-expressing cells, there was a shift to more slowly migrating forms with approximate molecular masses of 85–100 kDa. To rule out aberrant running of the SDS-PAGE gel as the cause of this electrophoretic shift, a second western blot was performed with fresh cell lysates (Fig. 6B, bottom). This blot more clearly indicated that a 75 kDa LAMP1 band that is present in pVHL-negative cell lysates was not seen in pVHL-expressing cells and a new 100 kDa band was apparently gained. It is likely that these changes in LAMP1 mobility represent altered glycosylation patterns.

## Discussion

In this report, the functional relationship between pVHL and PKCι was explored by knocking out PKCι expression in renal carcinoma cells, both without pVHL and with pVHL replaced. It was observed that PKCι, when present, has a role in increasing HIF-2α expression in a manner that is antagonistic to pVHL-mediated regulation of HIF-α. Conversely, PKCι knockout abrogated cellular properties that pVHL has been shown to influence, such as formation of tight junctions and downregulation of α5 integrin expression. Thus, there appears to be a complex relationship between the cellular functions of PKCι and pVHL.

It was observed that PKCι knockout led to decreased levels of HIF-2α in both pVHL-expressing 786-O and pVHL-null cells, making simple competition of HIF-α and PKCι for binding to pVHL’s beta domain not likely and instead supporting a notion that PKCι has a pVHL-independent role to increase HIF-α expression in cells. Interestingly, PKCζ had also been implicated previously in a pro-HIF-α role, as decreased transcriptional activity (but not abundance) of HIF-α was observed in 786-O cells with PKCζ knockdown or with a dominant-negative PKCζ [33]. Paralleling the decreased HIF-2α seen in PKCι knockouts, a slight decrease in HIF-2α levels was also observed herein when PKCζ was knocked out in pVHL-expressing RCC10 cells. Combined, these results could suggest that both isoforms of aPKC have a general function to promote HIF response in renal cells, perhaps by more than one mechanism. PKCι has been viewed as an oncogene (reviewed in [34]) and its role in increasing HIF-α reported here is consistent with this idea.

In contrast to a PKCι role in promoting cancer, PKCι was seen here to contribute to at least one cellular function that is shared with pVHL and is tumor inhibiting. Proper tight junctions oppose epithelial to mesenchymal transitions and are associated with a less metastatic phenotype. It is well established that proper aPKC function is needed for tight junction formation [19] and has also been shown that pVHL is necessary for tight junctions to form [22, 24, 25]. Given that loss of either PKCι or pVHL disrupts tight junctions, it is tempting to speculate that these two proteins act as part of the same complex toward this function based on their documented interaction [12, 14], although the data herein does not rule out that each has an independent effect.

Interestingly, in the presence of both pVHL and PKCι, decreased expression of α5 integrin was observed, and this decrease was reversed by lysosomal inhibition. Lack of pVHL caused a greater increase in α5 integrin levels than lack of PKCι, but loss of both did not have a synergistic effect. These data may suggest that pVHL and PKCι act in a common pathway regulating α5 integrin, but with distinct roles (and possibly without the need of a pVHL-PKCι complex). Integrins typically undergo cycles of endocytosis and recycling that affect their presence at the plasma membrane and ability to influence cell migration (reviewed in [35]). Integrins that are endocytosed can also end up in multivesicular endosomes and targeted for lysosomal degradation [30] and the data herein suggest that both PKCι and pVHL are necessary for this event to happen. However, loss of pVHL resulted in increased cellular motility, whereas loss of PKCι decreased motility. One possibility that fits with these findings is that PKCι is involved in the endocytosis of α5 integrin needed for α5 integrin recycling, whereas pVHL has a role in targeting, perhaps a specific pool, of endocytosed α5 integrin to the lysosome. Interestingly, aPKC has been shown to have a role in integrin endocytosis (reviewed in [36, 37]). This role of PKCι might lead to a pool of endocytosed α5 integrin that can be acted upon in some way by pVHL toward lysosomal degradation.

Although the most common heterodimer partner of α5 integrin is β1 integrin, forming the fibronectin receptor, knockout of PKCι did not have a consistent effect on the abundance of β1 integrin. Only one of the two PKCι 786-O knockout cell lines showed upregulated β1 integrin, although both showed a slight shift to the mature form. Moreover, upregulation of β1 integrin was only minimally observed in PKCι knockout RCC10 cells and in this cell line, the precursor form was increased (see Fig. 4A, 4th panel, lane 3). It is unclear why β1 integrin was not upregulated similarly as α5 integrin with PKCι loss, but this observation could be related to a higher relative abundance of β1 as compared to α5 integrin in renal carcinoma cells, as β1 integrin heteodimerizes more often with other α integrin subunits [38], which can be internalized by different mechanisms (reviewed in [36]). Of note, α5 integrin, but not β1 integrin, has been observed to be upregulated in cells treated with a lysosomal inhibitor [39], matching the findings here.

The specific molecular mechanism by which pVHL influences lysosomal degradation of α5 integrin is not immediately apparent, although pVHL roles in endocytosis have been previously described [40, 41]. pVHL has been shown to be needed for downregulation of activated EGFR via Rab5-mediated early endosome fusion in a manner that was dependent on pVHL regulation of HIF-α [40]. In contrast, pVHL has also been shown to be recruited to FGFR- (but not EGFR-) containing endosomes that internalize activated plasma membrane FGFR in a manner that was not affected by HIF-α [41]. With respect to the importance of pVHL regulation of HIF-α toward the endocytic phenomena observed here, the findings with FGFR endocytosis are more in agreement with the present observations, as regulation of integrin levels has been previously shown to be decoupled from pVHL-mediated degradation of HIF-α [42]. However, unlike the decrease in total cellular levels of α5 integrin seen here, only surface accumulation of FGFR, and not overall levels, was decreased by pVHL [41]. Thus, while there are commonalities among these pVHL-influenced endocytic events, it is likely that other specific influences on these different endocytic targets are at play. Also, it is unclear if lysosomal degradation is the predominant fate of all of these other endocytosed proteins, as shown for α5 integrin here.

Interestingly, a difference in localization and electrophoretic migration of the lysosomal protein, LAMP1, was seen in cells with and without pVHL. It is likely that the altered gel migration is due to a change in glycosylation of LAMP1, as glycosylation accounts for over 50% of the molecular mass of this protein [31]. LAMP1 has been seen to shift to more highly glycosylated forms due to cell differentiation [43, 44], thus the shift to slower LAMP1 migration observed here with pVHL expression, which has been shown to cause cell differentiation [18, 45], is in agreement. Changes in the glycosylation patterns of other proteins due to pVHL expression have been documented [46], thus it is possible that pVHL expression has a more global effect on glycosylation. It is possible that the presumed change in LAMP1 glycosylation is directly related to its altered localization. It is speculated that the observed changes in glycosylation and localization of LAMP1, especially the enlarged vesicles seen with pVHL-negative cells, is related to some functional impact on lysosomes and/or their biogenesis, although these results are preliminary and further investigation is warranted.

It remains to be determined whether pVHL ubiquitin ligase function is needed for the lysosomal targeting of α5 integrin and if so, what the target substrate is. One possibility that is neither implied nor excluded by this study is that pVHL interaction with and/or ubiquitination of PKCι plays a role in steering α5 integrin-containing endosomes to the lysosome. Interestingly, aPKCs can bind to p62/Sequestosome-1 (SQSTM1), which aids in delivering sequestered ubiquitinated proteins to the lysosome (reviewed in [47]), and it is conceivable that its interaction with pVHL somehow influences this function. Unfortunately, missense mutations in pVHL that disrupt complex formation with PKCι and would also maintain pVHL function have not been identified [12], making it difficult to sort out this possibility. Other substrates are also possible, including α5 integrin, for which ubiquitination has been seen to lead to its lysosomal degradation [30]. The sorting signal for lysosomal degradation of endocytosed membrane proteins can be multi-ubiquitination using a lysine 63 (K63) linkage [48–50]. Thus, it would be interesting to see if a pVHL-mediated K63 ubiquitination of α5 integrin or some other protein involved in the pathway is involved in the observed lysosomal degradation of α5 integrin. However, a role for pVHL in ubiquitination of PKCι while associated with α5 integrin-containing early endosomes, directing the endosome away from a recycling fate and toward the lysosomal degradation pathway, cannot be excluded by the data herein.

## Conclusions

In this study, a novel role of PKCι in upregulating HIF-α levels in a manner that was independent of pVHL and partly opposes pVHL-mediated downregulation of HIF-α was identified. Moreover, it was also demonstrated that both PKCι and pVHL are necessary for lysosomal degradation of α5 integrin in renal carcinoma cells, which is a novel role for pVHL that is suggested to occur downstream of PKCι-mediated endosomal internalization of α5 integrin. It is probable that regulation of α5 integrin lysosomal degradation by pVHL is important for its tumor suppressor functions, and additional studies are warranted.

## Electronic supplementary material

Below is the link to the electronic supplementary material.

Supplementary Material 1

## Data Availability

No datasets were generated or analysed during the current study.
