# Supplementary material for: Protein kinase C iota (PKCι) and pVHL are both needed for lysosomal degradation of α5 integrin in renal carcinoma cells
Source: Mol Biol Rep. 2025 Jan 30;52(1):177. doi: 10.1007/s11033-025-10272-1 (PMC11782342; doi:10.1007/s11033-025-10272-1)

# Fig. 1 (first 2 blots)

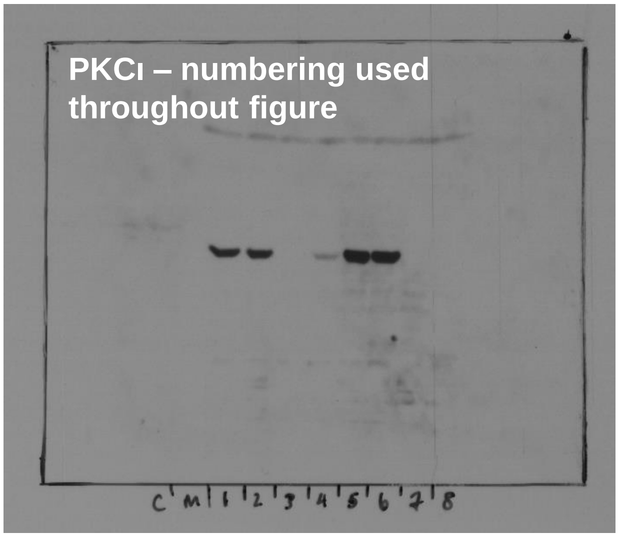


**top of blot labeled**

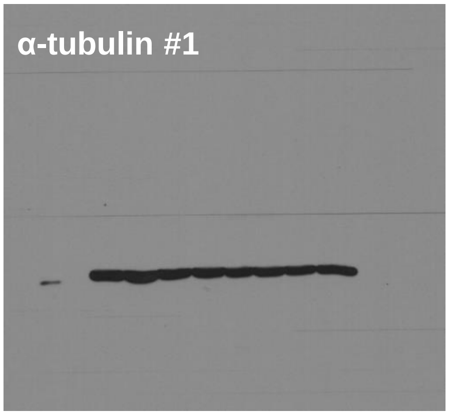


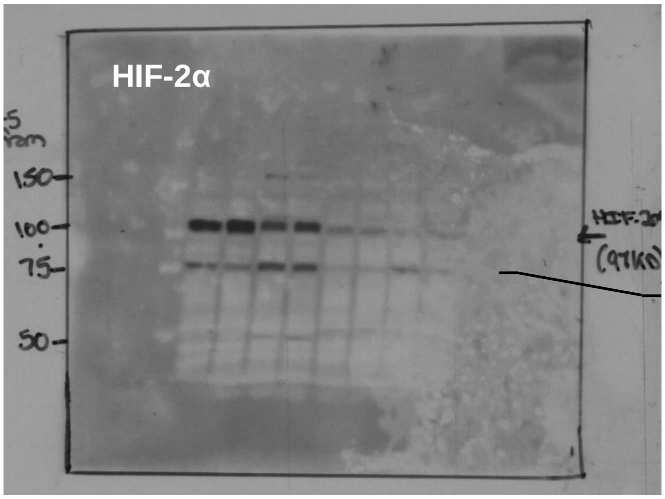


non-specific

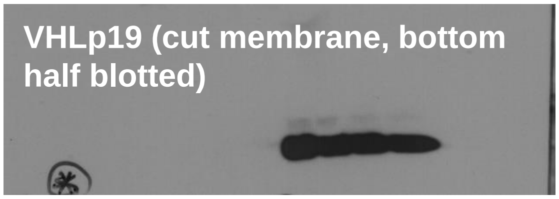


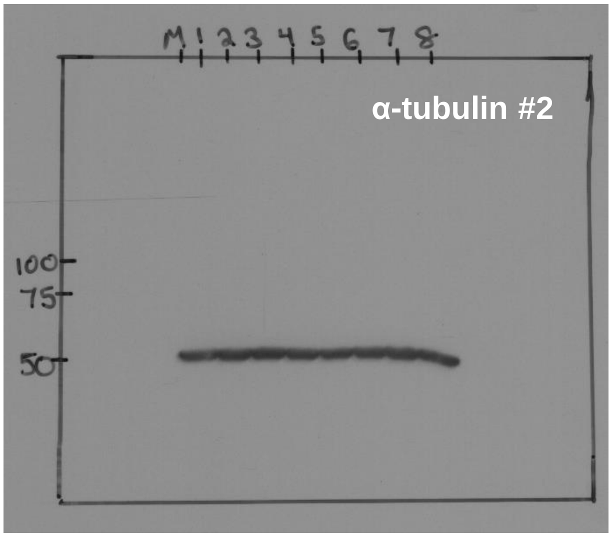

**Fig. 1 (3rd blot)**

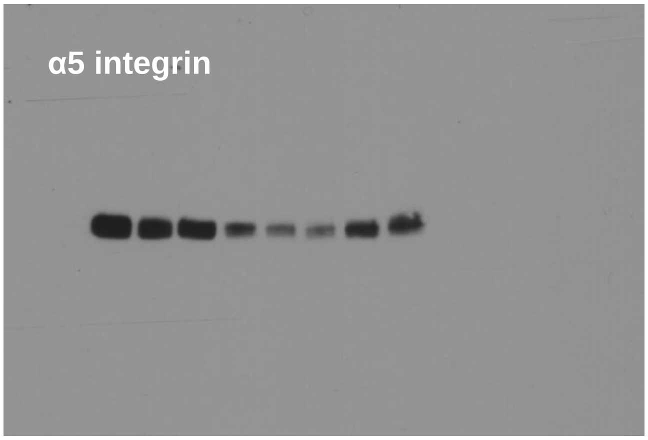


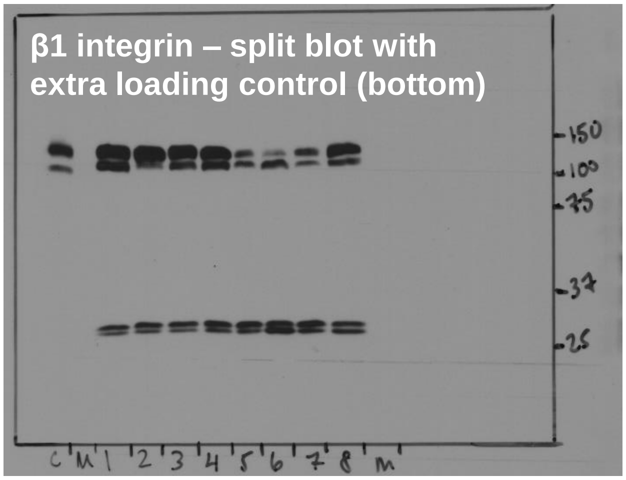


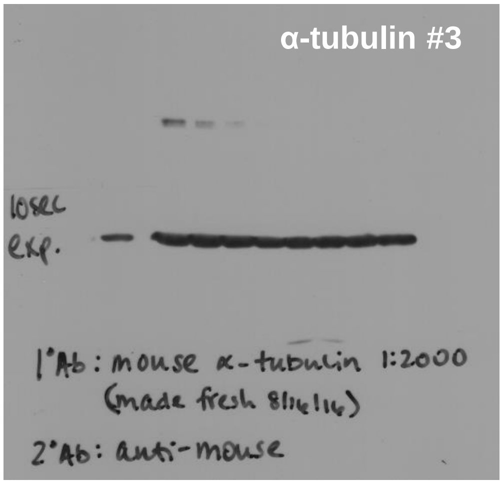

**Fig. 3a**

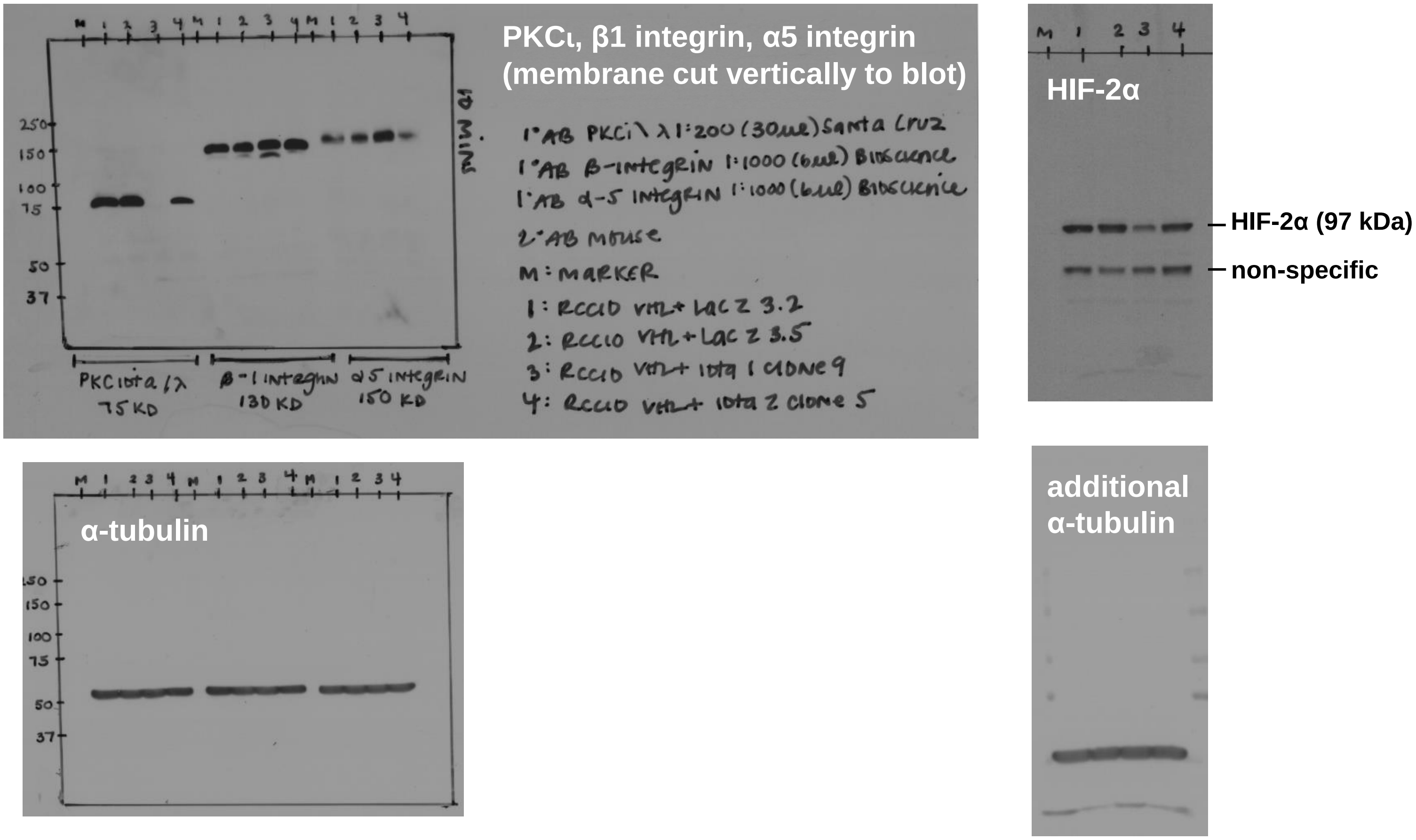
PKCι, β1 integrin, α5 integrin
(membrane cut vertically to blot)
HIF-2α
HIF-2α (97 kDa)
non-specific
α-tubulin
additional
α-tubulin

# Fig. 3b

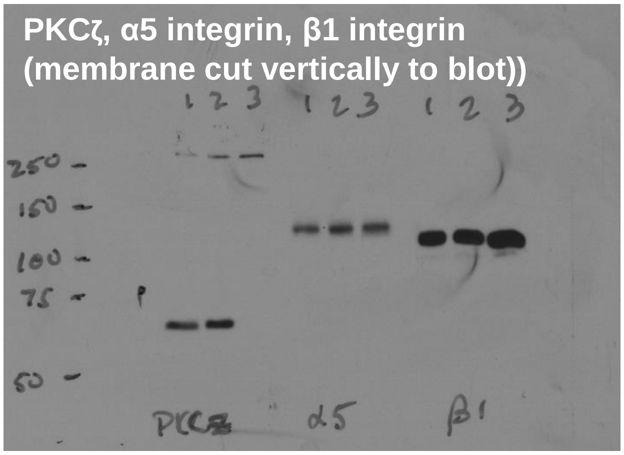


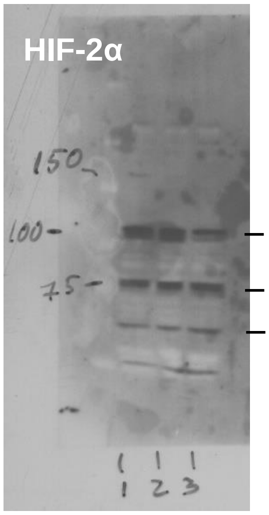


HIF-2α (97 kDa)

non-specific

non-specific

non-specific

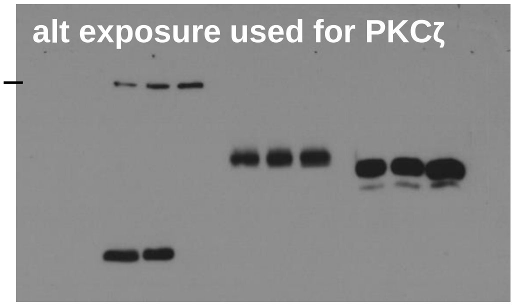


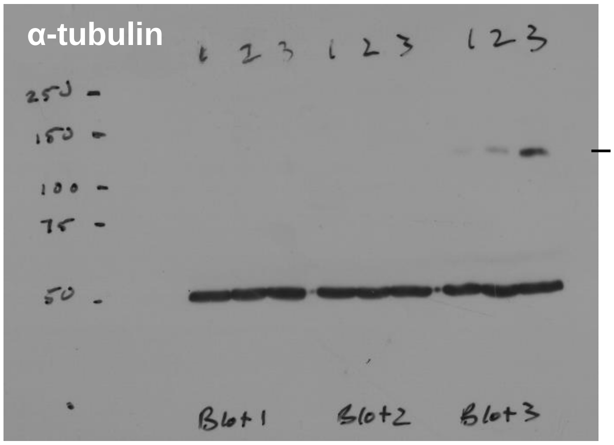


β1 integrin signal picked up in reblot

**Fig. 4a**

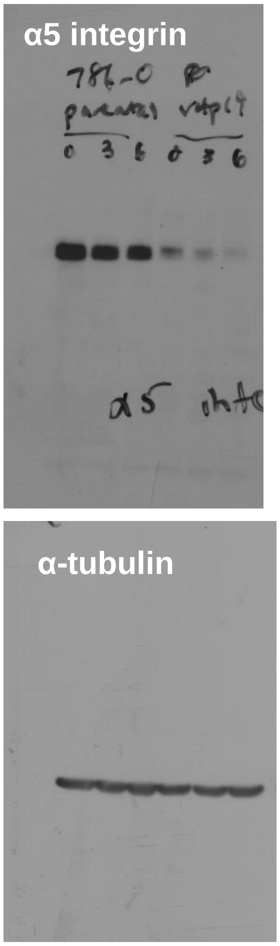


**Fig. 4b**

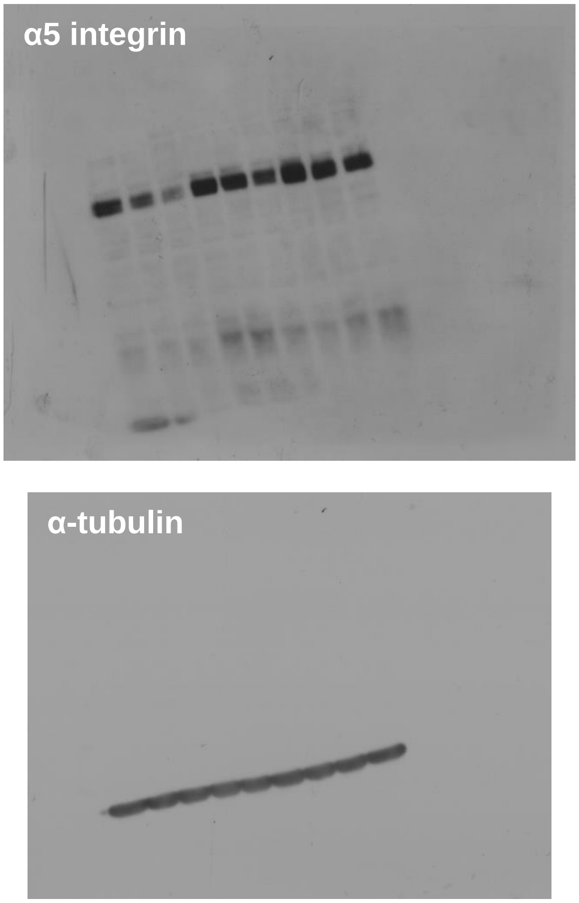


**Fig. 4c**

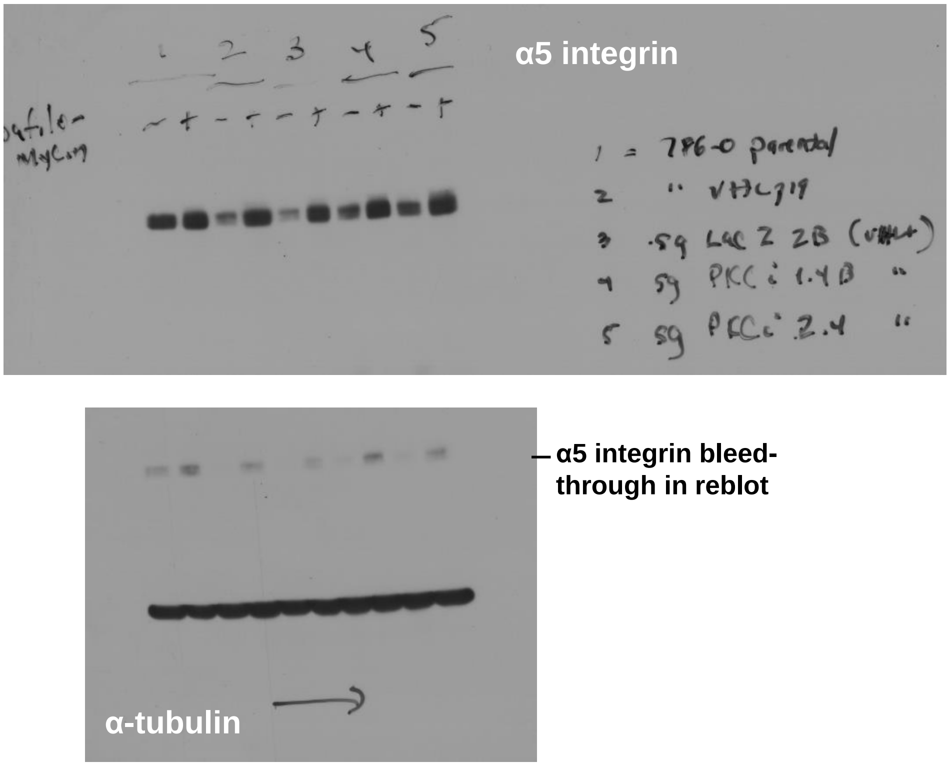

**Fig. 6b**

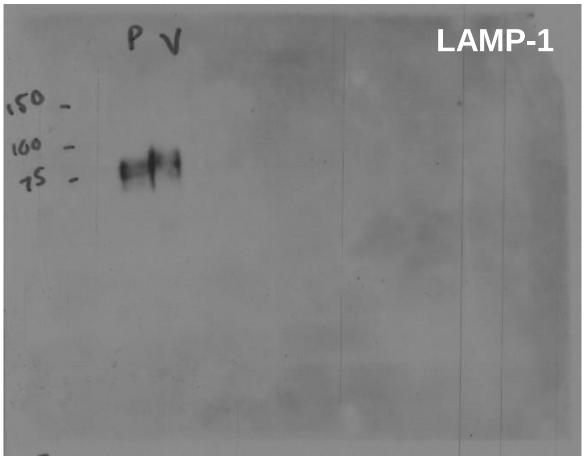


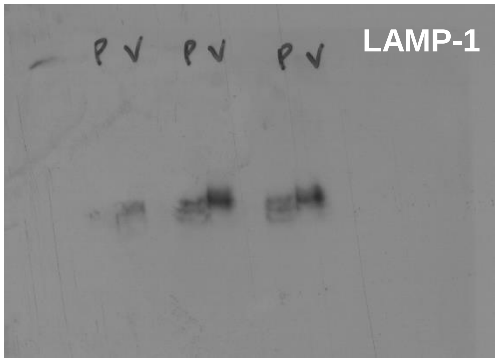


**note: additional lysates tested**

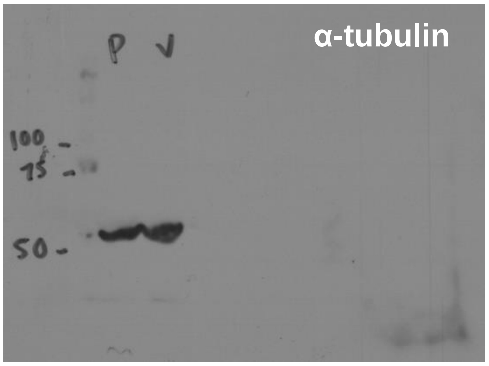


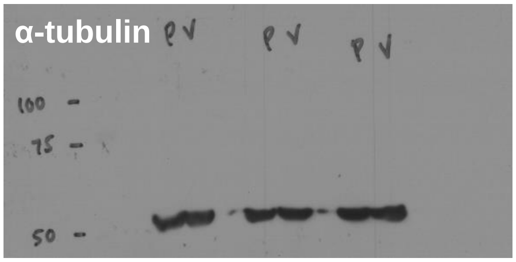

Supplement: Supplementary file 1 — Supplementary Material 1 [file 11033_2025_10272_MOESM1_ESM.pdf]
